# School bullying and minority identity as a menace to mental well-being of students in Greece

**DOI:** 10.1192/bji.2020.51

**Published:** 2021-08

**Authors:** Alexandra Gkouliama, Maria Samakouri, Aspasia Serdari

**Affiliations:** 1 Department of Child and Adolescent Psychiatry, Medical School, Democritus University of Thrace, Greece. Email: aserntar@med.duth.gr; 2 Department of Psychiatry, Medical School, Democritus University of Thrace, Greece

**Keywords:** School bullying, minority, mental health, multicultural, child psychiatry

## Abstract

Increasing migration and the resultant multiculturalism in Greek society has highlighted the importance of studying the role of school ethnic composition in bullying and peer victimisation, not least because ethnic minority students involved in bullying behaviours seem to experience high levels of internalising and externalising problems. It is imperative that schools work towards ensuring a safe environment for all students. This can be achieved through the implementation of policies that facilitate positive social interactions and address issues of bias-based bullying, thus contributing to social justice. This article examines school bullying and related policies in Greece in the context of increasing diversity and reviews their evolution in the past decade.

School bullying has been in the spotlight worldwide since 2000, when it was recognised by the World Health Organization as a matter of public health concern. Both bullying perpetration and victimisation are associated with adverse mental health outcomes and social vulnerability in adult life.^1^

Literature reveals that bullying is documented in different countries independently of their geographical, socioeconomic or cultural characteristics. A cross-national study^2^ indicated that 8.6–45.2% of children were frequently involved in school violence as victims, perpetrators or both. However, the prevalence of bullying and victimisation among countries varies; in Greece, reported rates of involvement in the phenomenon ranged from 26 to 41%, much higher compared with other countries.^2^

The aim of this article is to picture school bullying and related policies in Greece in the context of increasing diversity and to review their evolution in the past decade.

## Bullying in Greek schools

Greece is a south-eastern European country, located on the tip of the Balkan Peninsula. The problem of school bullying and victimisation seems to have started to concern Greek society later than other European countries. Related research has been small-scale, methodologically diverse and without any official institutional supervision, and associated literature was scarce until 2000;^3^ thus, drawing safe conclusions about the situation in Greece was difficult. In the past decade, the economic crisis has led to significant changes in the country's population composition. Greek society was believed to be rather traditional and homogeneous for a long period of time. Ethnocultural diversity in Greece began to increase since 1990, after the first massive settlement of citizens of the former USSR and economic immigrants from low-income countries of Eastern Europe. At present, Greece is also being forced to accept sweeping changes due to migration waves of asylum seekers from the Middle East. There are no official data about the precise number of refugee children in Greece nor the size of the population of ethnic, linguistic and religious minorities.^4^ It has been recently estimated that 18 500 refugee and migrant children reside in Greece,^5^ a significant number of whom have enrolled in public education.^6^

National health planning, complying with the United Nations Convention on the Rights of the Child, has included reception and accommodation services for refugees, in order to enhance respect for ‘otherness’. As far as educational policies are concerned, it is noteworthy that embracing children from ethnic minorities is hampered by language barriers and the difficulty of reliably assessing their academic needs. Nearly two decades ago, a national programme with international schools and reception classes set the aim of supporting ethnic minority children by providing a safe school milieu tailored to their special educational needs and this resulted in an increased enrolment of refugee and immigrant children in school (Greek Parliament Law 2413/96). These strategies targeted reducing social exclusion and preventing racist behaviour in the school environment.

Within the time frame of the recent economic crisis and the above-mentioned migration waves, the question of possible association of school multiculturalism with bullying behaviours was raised. Surveys conducted by the University Research Institute of Mental Health between 1998 and 2018 revealed that school bullying has significantly increased in the past decade compared with the early 2000s. Victimisation ranged between 19.1 and 28.9% of all school children, and being a bully was reported in 9.1% of these children in 2002, 15.8% in 2010 and 14.2% in 2018.^7^ According to a survey in six European countries (Greece, Italy, Bulgaria, Estonia, Latvia and Lithuania), about 42% of teachers believed that instances of bullying and violence in schools were often downplayed or covered up by parents or school staff, who denied involvement of their child or their school respectively.^8^

According to Stavrou et al,^7^ 3.9% of adolescents in Greece reported being victimised because of their sexual identity, 2.8% because of their origin and 2.1% because of their family's socioeconomic status. The most frequent form of bullying was verbal violence, such as spreading false rumours and sexual insults. Boys reported more often experiencing physical violence and being insulted because of their ethnic identity or religion, whereas girls seemed to suffer mainly from false rumours.

## School bullying and minority identity

Bullying behaviours are related to intolerance towards anything diverse or unfamiliar regarding norms, identities and attitudes; therefore, ethnicity may function as a status characteristic contributing to peer victimisation.^9^ This raises questions on whether discriminatory bullying may be attributed to the children's ‘immigrant status’ and be affected by the composition of the school population.^10^

A noteworthy example that has allowed the study of school bullying in relation to cultural identity in Greece is the geographical subdivision of Thrace. It is the north-eastern prefecture of the country, bordering Bulgaria and Turkey. Thrace's cultural diversity has been formed by various ethnolinguistic and religious groups over the years. Its population consists of (a) the Christian Orthodox population; (b) the Muslim minority, which is a dominant minority group, including the Pomaks and the Roma people; and (c) the descendants of Armenian refugees and expatriated Greeks from countries of the former Soviet republics settled in Thrace after 1990.^11^ All these cultural minorities are socially introverted and retain many of their customs. According to research on school bullying behaviours in Thrace,^12^ one in three secondary school students has been involved in bullying behaviours, nearly a quarter were identified as victims and one in six reported having bullied others. Students of minority status reported having experienced discriminatory victimisation more frequently compared with the majority group. School size was a crucial factor; schools with fewer students were less likely to record bullying incidents, compared with schools with a large student population. Additionally, both aggression and victimisation were more frequently observed when minority and majority students’ groups were almost numerically equal. On the contrary, peer victimisation was less frequent in schools where the minority group was either outnumbered or predominant compared with the majority students. These results could be explained by Blalock's group threat theory, which posits that feelings of ethnic threat are more intense in contexts where the different ethnic groups are of roughly equal size.^13^

## School bullying and children's mental health

Children and young people involved in bullying are very often engaged in risky behaviours, present with low academic achievement and experience mental health difficulties that accompany them into adulthood.^14^ Adolescents from ethnic minority groups in particular who were involved in bullying behaviours reported more internalising problems compared with uninvolved peers.^12,15^

More specifically, it has been noted that victims of bullying frequently suffer from anxiety, depression, social withdrawal and various psychosomatic complaints, whereas bullying perpetrators demonstrate high levels of externalising problems, such as aggression and rule violation.^12,16^ Children of dual status (bully/victim) seem to be the most vulnerable group, usually reporting low self-esteem, extremely high rates of depression and anxiety, and school maladaptation compared with other involved children. They also present with hyperactivity and impulsiveness and experience peer rejection.^17^

As a result, fear of school and truancy are more prevalent among students involved in bullying behaviours compared with unaffected peers.^12,18^

## The challenge for a national policy

European countries have developed different strategies to target bullying. Many countries participate in international anti-bullying actions funded by the European Union (e.g. Ireland, Italy, Lithuania, Romania), while others have adopted integrated national policies implemented by their educational authorities (e.g. Bulgaria, France, Romania, the UK). Sweden, Belgium and Malta have also enacted anti-bullying laws to implement practices against school violence.^19^

In Greece, a national policy against bullying has been relatively recently adopted. In 2012, the Hellenic Ministry of Education formed an Observatory for the Prevention of School Violence and Bullying. It plans and coordinates the anti-bullying prevention strategy, mainly based on enhancing relative awareness among teachers and evaluating school programmes, with the aim of facilitating positive social interactions among all students. Furthermore, school counsellors, education directors and teachers were called to raise bullying mindfulness among students and invest time in extra-curricular activities. A telephone helpline has also been set up by a non-governmental organisation to provide confidential out-of-hours support, crisis resolution and health-specialist advice. Public opinion has been significantly sensitised thanks to parental psychoeducation. March 6 was established as a National Day of Action against School Violence.

Policies addressing the educational integration of migrant children and the management of cultural diversity include intercultural schools, reception classes, educational priority zones, amplifier tutorials and foreign schools. The ultimate goal is to prevent school violence, through social integration.

School bullying and its impact on mental health demands an interdisciplinary approach and public health initiatives, including culturally sensitive anti-bullying campaigns.

## Supplementary material

For supplementary material accompanying this paper visit https://doi.org/10.1192/bji.2020.51. click here to view supplementary material
